# Sex-Specific Temporal Patterns of Comorbid Mental, Behavioral, and Somatic Diagnoses Relative to Substance Use Disorder Onset: Evidence from the Norwegian Patient Registry

**DOI:** 10.1192/j.eurpsy.2026.10483

**Published:** 2026-07-31

**Authors:** M. Scarth, A. Bjørnebekk, M. Riksheim Stavseth, J. G. Bramness

**Affiliations:** 1 Oslo University Hospital; 2 University of Oslo, Oslo; 3 UiT The Arctic University of Norway, Trondheim; 4 Innlandet Hospital Trust, Hamar, Norway

## Abstract

**Introduction:**

Substance use disorders (SUDs) are often comorbid with mental and somatic conditions, and the relationship between these diagnoses and SUD onset may differ by sex. Understanding which diagnoses more often precede or follow SUDs, and how these patterns vary by SUD type and sex, can help identify risk factors and inform prevention strategies.

**Objectives:**

This study aims to describe the prevalence of mental/behavioral and somatic diagnoses before and after SUD diagnosis in the Norwegian Patient Registry (NPR), examine sex differences in timing, and compare patterns across SUD types, including alcohol, opioid, and cannabis use disorders.

**Methods:**

We included individuals with a SUD diagnosis between 2008–2016 from the NPR. A 2-year washout period was applied to approximate the first SUD diagnosis. Diagnoses were based on ICD-10 codes, categorized by chapter, with mental and behavioral diagnoses categorized into more specific clinically relevant groups. SUD type was determined by whether an individual had only AUD, only OUD, only CUD, multiple SUDs, or other SUDs. Time between the first SUD and comorbid diagnoses was calculated in months before, after, or within the same month.

**Results:**

We identified 65,743 individuals with SUD diagnoses (35% female). The most common somatic diagnosis preceding SUD was musculoskeletal disorders, particularly in OUD (24.4% at 24+ months before). Depression was the most common mental diagnosis, with higher prevalence after the first SUD diagnosis in CUD only and multiple SUDs (10% in both groups at 1–12 months after SUD diagnosis). Sex differences showed that women with OUD had musculoskeletal disorders more frequently before the OUD diagnosis relative to men. Depression was more frequently recorded after the first SUD for both sexes with AUD or multiple SUDs. Among women with CUD or OUD, depression was more often diagnosed before SUD onset.

**Conclusions:**

Our findings highlight distinct temporal patterns of comorbid diagnoses relative to SUD onset, with musculoskeletal disorders preceding OUD and depression frequently following AUD or multiple SUDs. Sex-specific differences, especially among women with CUD or OUD, suggest that the timing of comorbid diagnoses varies by both SUD type and sex.

**Disclosure of Interest:**

None Declared

